# Community Infections Linked with Parvovirus B19 Genomic DNA in Wastewater, Texas, USA, 2023–2024

**DOI:** 10.3201/eid3107.241981

**Published:** 2025-07

**Authors:** Alessandro Zulli, Rebecca Y. Linfield, Dorothea Duong, Bridgette Hughes, Alexandria B. Boehm

**Affiliations:** Stanford University, Stanford, California, USA (A. Zulli, R.Y. Linfield, A.B. Boehm); Verily Life Sciences LLC, South San Francisco, California, USA (D. Duong, B. Hughes)

**Keywords:** parvovirus B19, viruses, community infections, hydrops fetalis, wastewater, Texas, slapped cheek rash, wastewater surveillance, United States

## Abstract

We assessed concentrations of parvovirus B19 DNA from 2 wastewater treatment plants in a Texas, USA, county with a known outbreak in 2024. Wastewater viral concentrations correlated significantly with clinical cases, demonstrating wastewater’s potential for tracking parvovirus B19 infections. Peaks in wastewater concentrations were aligned with the peak in hydrops fetalis diagnoses.

Parvovirus B19 is a single-stranded DNA virus transmitted through respiratory droplets. In 2024, infections increased substantially across the United States; seroprevalences in children rose from <3% in 2020–2022 to 24.9% in 2024 (*1*). Parvovirus B19 typically causes a mild slapped cheek rash in children but can lead to serious complications in pregnant women, including hydrops fetalis and fetal death (*2*–*4*). Persons infected with the virus, particularly those who are immunocompromised, are at risk for developing aplastic anemia (*3*,*5*).

Parvovirus B19 is highly infectious, with an estimated basic reproduction number (R_0_) of 8, and studies have shown that 20%–50% of susceptible persons are infected during outbreaks in schools (*6*,*7*). No vaccine is available for the virus and treatment options are limited, meaning prevention and early detection are crucial (*1*,*4*,*8*). Nonetheless, parvovirus B19 infection is not a notifiable condition to public health authorities in the United States (*6*). This lack of surveillance led to a Health Alert Network notice from the Centers for Disease Control and Prevention in August 2024, months after the initial rise in cases had occurred (*6*). 

Wastewater-based epidemiology has been shown to be an effective, real-time, and low-cost method of generating epidemiologic data for a variety of human pathogens, suggesting potential utility for parvovirus B19 (*9*,*10*). Our study aimed to evaluate whether wastewater surveillance can track parvovirus B19 infections by analyzing samples from 2 wastewater treatment plants (WWTPs) during a known outbreak in Montgomery, Texas, USA, and comparing results with clinical information from electronic health records systems. Our study was reviewed by the Stanford University Committee for the Protection of Human Subjects and determined to be exempt from oversight.

## The Study

We collected 24-hour composite raw wastewater influent samples 3 times/week during December 18, 2023–August 30, 2024, from 2 treatment plants serving 65,000 (WWTP186) and 70,000 (WWTP187) persons (Appendix Figure 1). We collected a total of 220 samples. From each sample, we collected wastewater solids using settling and centrifugation.

We used a previously validated hydrolysis-probe PCR targeting the nonstructural protein 1 gene of parvovirus B19. We confirmed assay specificity and sensitivity through in silico analysis against reference genomes and in vitro testing against common respiratory viruses and bacteria (Appendix, Table 1). We extracted DNA from wastewater solids using the chemagic Viral DNA/RNA 300 kit (Revvity, https://www.revvity.com), followed by column-based PCR inhibitor removal. We quantified viral concentrations of parvovirus B19 (fluorescent molecule FAM) and SARS-CoV-2 (fluorescent molecule HEX) as a duplex assay in 6 replicate wells using digital droplet reverse transcription PCR (Appendix Table 2). We included negative and positive extraction and PCR controls. The lowest detectable concentration was ≈1,000 copies/g of dry weight. Complete methods, including primers and thermocycling conditions and further details of various controls, are provided (Appendix). Measured concentrations in the samples are available through the Stanford Digital Repository (https://doi.org/10.25740/zn011jk5743).

We gathered clinical case and syndromic data from Epic Cosmos (Epic Systems Corporation, https://cosmos.epic.com). First, we filtered all encounters in the dataset geographically and temporally to encounters within Montgomery County, Texas, during October 16, 2023–October 16, 2024. Montgomery County had a total population of 620,443 as of the 2020 census. From this subset, we identified parvovirus cases and hydrops fetalis diagnoses using grouped codes from the International Classification of Diseases, 10th Revision (ICD-10) (*11*). We identified parvovirus cases using ICD-10 codes B08.3, B34.3, and B97.6, and hydrops fetalis using ICD-10 codes P56.*, O36.2*, and Z36.81. We aggregated data weekly for parvovirus cases and quarterly for hydrops fetalis diagnoses; we redacted values <10 and replaced with 10 (Appendix).

Our statistical analyses used Kruskal-Wallis tests to compare viral concentrations between plants and χ^2^ tests for detection frequencies. We assessed associations between wastewater concentrations and clinical cases using Kendall tau correlation. Because we made multiple comparisons (n = 11), we used p = 0.005 as the significance threshold.

Our analyses detected parvovirus B19 DNA in 45 (40%) of 111 samples from WWTP186 and in 62 (57%) of 109 samples from WWTP187; detection frequencies were not significantly different (χ^2^= 5.24; p = 0.02). Concentrations were mostly nondetectable before April, peaked in June, and returned to nondetectable levels by August. Median (interquartile range [IQR]) concentrations were 0 (IQR 0–8,071 copies/g) at WWTP186 and 6,121 (IQR 0–20,998 copies/g) at WWTP187, showing significant differences between plants (effect size = 6,121 copies /g; p = 0.001) even when normalized by pepper mild mottle virus (effect size = 2.7 × 10^−5^, p = 0.003).

Clinical parvovirus cases in Montgomery County showed similar seasonality: ≤10 cases until April, a peak in June, and a decline by August. Weekly viral concentrations correlated significantly with case counts at both plants (Kendall tau = 0.56 at WWTP186 and 0.52 at WWTP187; both p<0.0001). During the quarter of increasing wastewater concentrations, parvovirus cases rose from <10 to 223, coinciding with hydrops fetalis cases increasing to 49 (Figure).

**Figure:**
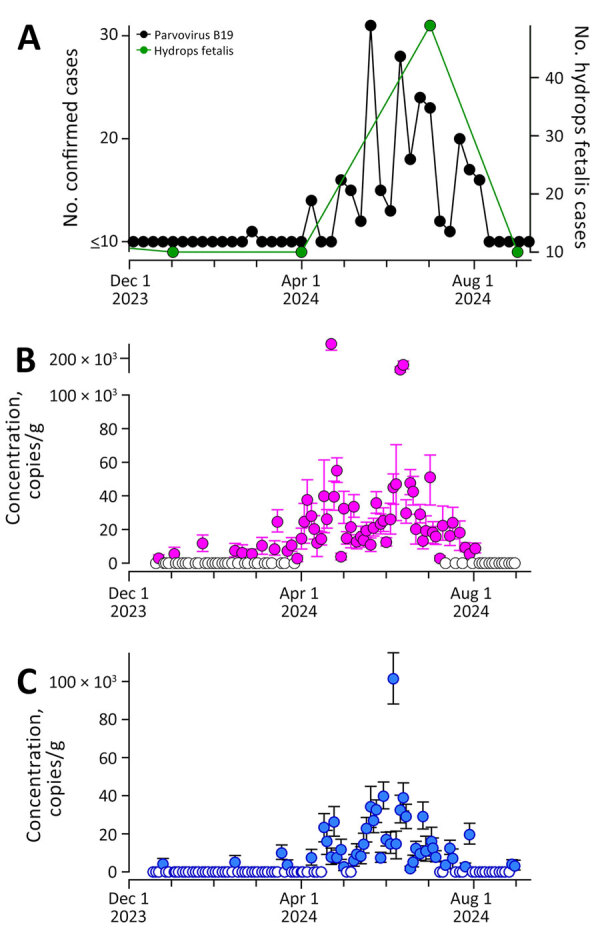
Comparison of case reports and wastewater surveillance reports from study of community infections linked with parvovirus B19 genomic DNA in wastewater, Montgomery County, Texas, USA, 2023–2024. A) Numbers of confirmed parvovirus infections and hydrops fetalis logged into Epic Cosmos (https://cosmos.epic.com) for the county. Data on fetal hydrops were provided quarterly; parvovirus infection data were provided weekly. Scales for the y-axes differ substantially to underscore patterns but do not permit direct comparisons. B, C) Concentrations of parvovirus B19 DNA in wastewater solids (in units of copies/gram of dry weight) at WWTP186 (B) and WWTP187 (C). Solid circles indicate parvovirus B19 was detected, open circles that it was not detected; error bars indicate SDs. Note the broken y-axis in panel B. For the case and syndromic data, numbers ≤10 are redacted and reported as 10. Kendall tau between weekly cases and wastewater concentrations for WWTP186 was 0.56 and between weekly cases and wastewater concentrations for WWTP187 was 0.52 (p<0.0001). WWTP, wastewater treatment plant.

## Conclusions

Our study demonstrates that parvovirus B19 DNA can be quantified in wastewater and directly correlated with clinical indicators of infection. Our results validate the potential for wastewater to provide sentinel surveillance during key early periods of an outbreak, especially in the absence of mandated public health reporting. Previous studies have shown that parvovirus B19 infects the intestinal mucosa and is present in the sputum of infected patients, indicating it could be shed into wastewater (*12*). Investigators have shown related viruses, such as human bocaviruses, to be shed in stool samples (*13*). Recent studies have identified parvovirus B19 (often referred to as erythroparvovirus) in wastewater samples through shotgun sequencing and metagenomic analysis (*14*,*15*).

Parvovirus B19 DNA concentrations in wastewater at both treatment plants were significantly correlated with clinical cases of parvovirus B19 in the county. Most (>90%) of those diagnosed cases were in children and adolescents <16 years of age, likely because of the unique symptomatic presentation in children as a slapped cheek rash and clinical diagnosis without further molecular diagnostics. We also showed that B19 DNA concentrations in wastewater at both treatment plants corresponded to an increase in maternal care for hydrops fetalis, one of the known complications of parvovirus B19 in pregnant women. This finding suggests that parvovirus B19 DNA in wastewater is an indicator of not only infections in children, but infections in the adult population, for which clinical data are highly limited. 

In summary, wastewater represents a rapid and cost-effective method of providing real-time information on parvovirus B19 outbreaks in populations. This early detection is especially important because children are no longer viremic by the time the characteristic rash appears. Proactive surveillance of wastewater to detect viremia could aid in mitigation strategies, such as warning or screening pregnant women and those who are immunocompromised.

Appendix Additional information for community infections linked with parvovirus B19 genomic DNA in wastewater, Texas, USA, 2023–2024.
